# Synthesis of Autofluorescent Phenanthrene Microparticles via Emulsification: A Useful Synthetic Mimic for Polycyclic Aromatic Hydrocarbon-Based Cosmic Dust

**DOI:** 10.1021/acsami.3c08585

**Published:** 2023-11-09

**Authors:** Derek H. H. Chan, Jessica L. Wills, Jon D. Tandy, Mark J. Burchell, Penelope J. Wozniakiewicz, Luke S. Alesbrook, Steven P. Armes

**Affiliations:** † Dainton Building, Department of Chemistry, University of Sheffield, Brook Hill, Sheffield, South Yorkshire S3 7HF, U.K.; ‡ School of Physics and Astronomy, University of Kent, Canterbury, Kent CT2 7NH, U.K.; § School of Chemistry and Forensic Science, University of Kent, Canterbury, Kent CT2 7NZ, U.K.

**Keywords:** phenanthrene, polycyclic aromatic hydrocarbons, polypyrrole, cosmic dust, synthetic mimics, emulsification

## Abstract

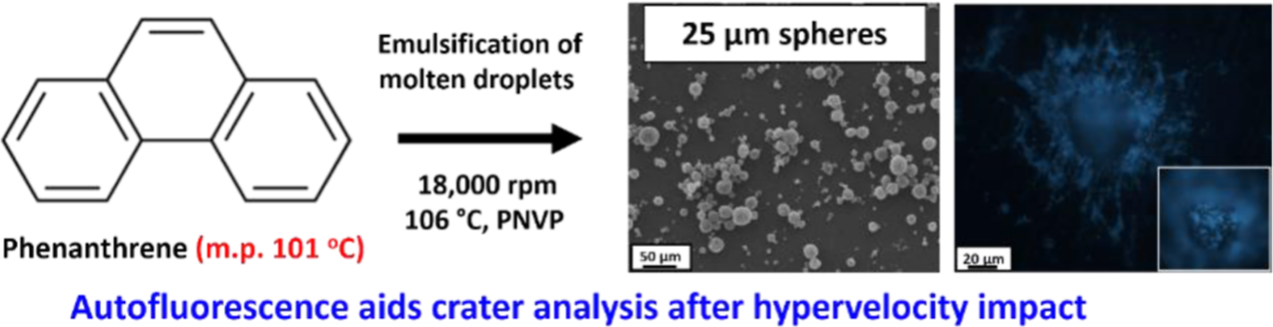

Phenanthrene is the simplest example of a polycyclic aromatic hydrocarbon (PAH). Herein, we exploit its relatively low melting point (101 °C) to prepare microparticles from molten phenanthrene droplets by conducting high-shear homogenization in a 3:1 water/ethylene glycol mixture at 105 °C using poly(*N*-vinylpyrrolidone) as a non-ionic polymeric emulsifier. Scanning electron microscopy studies confirm that this protocol produces polydisperse phenanthrene microparticles with a spherical morphology: laser diffraction studies indicate a volume-average diameter of 25 ± 21 μm. Such projectiles are fired into an aluminum foil target at 1.87 km s^–1^ using a two-stage light gas gun. Interestingly, the autofluorescence exhibited by phenanthrene aids analysis of the resulting impact craters. More specifically, it enables assessment of the spatial distribution of any surviving phenanthrene in the vicinity of each crater. Furthermore, these phenanthrene microparticles can be coated with an ultrathin overlayer of polypyrrole, which reduces their autofluorescence. In principle, such core–shell microparticles should be useful for assessing the extent of thermal ablation that is likely to occur when they are fired into aerogel targets. Accordingly, polypyrrole-coated microparticles were fired into an aerogel target at 2.07 km s^–1^. Intact microparticles were identified at the end of carrot tracks and their relatively weak autofluorescence suggests that thermal ablation during aerogel capture did not completely remove the polypyrrole overlayer. Thus, these new core–shell microparticles appear to be useful model projectiles for assessing the extent of thermal processing that can occur in such experiments, which have implications for the capture of intact PAH-based dust grains originating from cometary tails or from plumes emanating from icy satellites (e.g., Enceladus) in future space missions.

## Introduction

Cosmic dust is ubiquitous throughout the Solar System: sources include asteroid collisions,^1^ cometary tails,^2^ volcanic plumes erupting from Saturn’s moons (e.g., Enceladus),^3^ and the interstellar wind.^4^ Such micrometeorites typically travel at speeds within the hypervelocity regime (>1 km s^–1^).^5^ Of particular relevance to the present study, cosmic dust particles comprising polycyclic aromatic hydrocarbons (PAHs) have been suggested as a potential signature for extant life. They have been detected in both terrestrial and Martian meteorites,^6^ in interplanetary dust,^7^ in the upper atmosphere of Titan,^8^ and in cometary tails.^9^ Moreover, one of the main objectives of the ESA ExoMars rover mission (now planned for a 2028 launch) is to search for PAHs within the Martian subsoil using a portable capillary electrophoresis instrument known as the Mars Organic Analyzer.^10^

**Figure 1:**
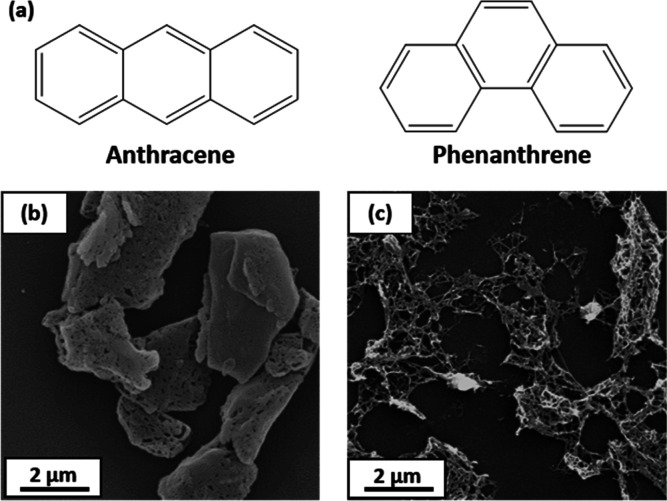
(a) Chemical structures of anthracene and phenanthrene; (b) scanning electron microscopy (SEM) image recorded for anthracene microparticles prepared via wet ball milling (left);^11^ and (c) SEM image recorded for phenanthrene after wet ball milling (right).

Recently, we reported the first synthetic mimic for PAH-based cosmic dust.^11^ Anthracene (C_14_H_10_) was prepared in the form of micrometer-sized particles by wet ball milling. This processing technique has been utilized for several decades for the industrial-scale preparation of aqueous suspensions of water-insoluble crystalline agrochemical actives such as azoxystrobin.^12,13^ Despite their rather ill-defined non-spherical morphology (see Figure 1),^11^ the resulting anthracene microparticles were fired at 1.87 km s^–1^ into an aluminum foil target^11^ using a two-stage light gas gun.^14^ Alternatively, they can be coated with an ultrathin overlayer of an electrically conductive polymer (polypyrrole or PPy).^11^ It is well-known that such coatings enable the efficient accumulation of surface charge, which should enable electrostatic acceleration of these anthracene microparticles up to the hypervelocity regime using a high-voltage van de Graaff accelerator.^14−20^ Impact ionization occurs when such fast-moving projectiles impinge on metal targets and the resulting ionic plasma can be analyzed by time-of-flight (ToF) mass spectrometry.^14−20^ Indeed, this is the basis for the detection of cosmic dust by instruments such as the Cosmic Dust Analyzer (CDA) on the CASSINI spacecraft.^21^ Laboratory-based experiments conducted with suitable synthetic mimics of known chemical composition were essential for calibration of the CDA, which facilitated interpretation of its cosmic dust data.^17,22^

Phenanthrene and anthracene comprise the two simplest examples of PAHs: they are structural isomers with an identical atomic mass of 178.23 g mol^–1^ (see Figure 1). Interestingly, recent literature^23−25^ suggests that these two compounds may exhibit subtly different mass fragmentation patterns during impact ionization when impinging on metal targets at >1 km s^–1^. Our preparation of micron-sized anthracene microparticles via wet ball milling was conducted in the presence of Morwet D-425, a commercially available anionic water-soluble polymer (see Figure 1b).^11^ However, when the same wet ball-milling protocol was employed for phenanthrene, only ill-defined microparticles exhibiting an unusual weblike morphology were obtained (see Figure 1c).

**Scheme 1:**
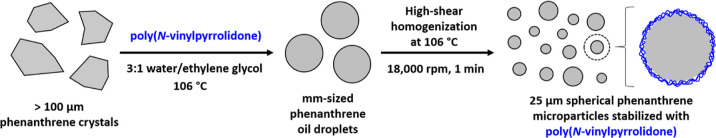
Schematic Cartoon for the Preparation of Phenanthrene Microparticles via High-Shear Emulsification of Molten Phenanthrene in a 3:1 v/v Water/Ethylene Glycol Mixture at 106 °C

**Figure 2:**
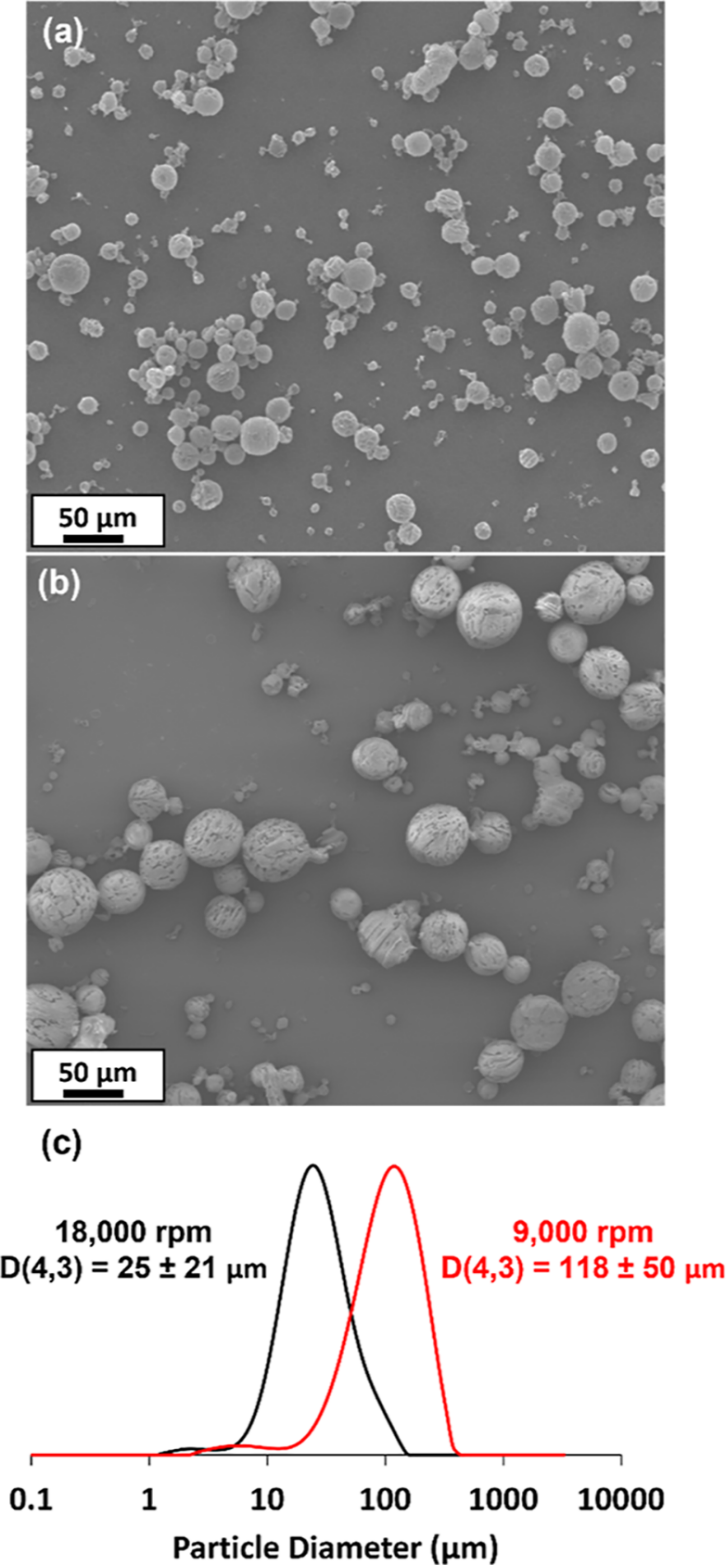
Representative SEM images obtained for phenanthrene microparticles prepared at (a) 18,000 rpm and (b) 9000 rpm via high-shear emulsification of molten phenanthrene. (c) Typical particle size distribution obtained for phenanthrene microparticles as determined by laser diffraction.

Unfortunately, such fragile projectiles tend to break up when accelerated up to hypervelocities. Seeking an alternative approach to wet ball milling, in the present study we take advantage of phenanthrene’s relatively low melting point (101 °C vs 216 °C for anthracene). Accordingly, coarse phenanthrene crystals are heated up to 106 °C in a 3:1 v/v water/ethylene glycol mixture (see Scheme 1). The resulting hot oil is then homogenized under high shear in the presence of a commercial water-soluble polymeric emulsifier, poly(*N*-vinylpyrrolidone), to produce molten micrometer-sized phenanthrene droplets. Polydisperse spherical phenanthrene microparticles are obtained on cooling to 20 °C (Figure 2). Such projectiles can be employed as projectiles for hypervelocity experiments conducted using a two-stage light gas gun^14^ and an aluminum foil target.

## Experimental Section

### Materials

Phenanthrene (98%) was purchased from Thermo Scientific (UK). Ammonium persulfate (APS, 98%), poly(*N*-vinylpyrrolidone) (nominal molecular weight = 360,000 g mol^–1^), ethylene glycol (anhydrous, 99.8%), and pyrrole (98%) were purchased from Sigma-Aldrich (UK). Pyrrole was purified by alumina chromatography (basic alumina, Sigma-Aldrich UK) prior to use. Deionized water obtained from an Elga Medica DV25 unit was used for all experiments.

### Preparation of Spherical Phenanthrene Microparticles

Phenanthrene (2.00 g, 5.0% w/w), poly(*N*-vinylpyrrolidone) (0.40 g, 1.0% w/w), deionized water (28.20 g), and ethylene glycol (9.40 g) were added to a 100 mL two-neck round-bottomed flask. A Findenser air condenser was attached to this flask and the second inlet was sealed using a rubber septum. The flask was immersed in an oil bath set at 120 °C. The phenanthrene crystals subsequently melted, leading to the formation of large (mm-sized) oil droplets. An IKA ULTRA-TURRAX T-18 homogenizer equipped with a 10 mm dispersing tool was lowered into the flask until the dispersing tool head was completely covered. High-shear homogenization of the oil–water mixture at a stirring rate of 6000 to 18,000 rpm was conducted for 1 min at 106 °C (a schematic cartoon of this experimental set-up is provided in the Supporting Information, see Figure S1). The resulting hot milky-white emulsion/suspension was immediately vacuum-filtered using a Buchner funnel and the moist white solid was quickly redispersed in deionized water (50 mL). The final milky-white suspension was freeze-dried to produce a fine white powder.

### Preparation of Polypyrrole-Coated Phenanthrene Microparticles

Phenanthrene microparticles obtained using the above protocol were subjected to three centrifugation–redispersion cycles (8000 rpm, 10 min per cycle) to remove excess poly(*N*-vinylpyrrolidone) emulsifier. For each cycle, the aqueous supernatant was carefully decanted, and the sediment was redispersed using fresh deionized water. The following protocol was used to coat 25 μm phenanthrene spherical microparticles with a target polypyrrole overlayer thickness of 30 nm. A 15% (w/w) aqueous suspension of phenanthrene microparticles (3.00 g; 0.45 g dry weight of phenanthrene) was stirred in a 10 mL glass vial at 800 rpm. Pyrrole (6.90 μL) was added to this vial followed by the addition of ammonium persulfate (13.1 mg; persulfate/pyrrole molar ratio = 0.58; target polypyrrole mass loading = 0.9%) dissolved in deionized water (0.25 mL). The initial milky-white suspension turned black during magnetic stirring for 30 min at 20 °C. The resulting polypyrrole-coated phenanthrene microparticles were purified by three centrifugation–redispersion cycles (8000 rpm, 10 min) to remove unreacted pyrrole and spent oxidant, followed by freeze-drying from water overnight to afford a fine, dark gray powder.

### Solvent Extraction of Phenanthrene from Polypyrrole-Coated Phenanthrene Microparticles

An aqueous suspension of polypyrrole-coated phenanthrene microparticles was dried onto a 1 × 1 cm silicon wafer to afford a dark gray powder. This wafer was then dipped into acetone (5 mL) and left in contact for 30 s at 20 °C. This protocol was repeated three times using fresh acetone in each case to produce a black residue for SEM analysis.

### Laser Diffraction Particle Size Analysis

Phenanthrene microparticles were analyzed using a Malvern Mastersizer 3000 laser diffraction instrument equipped with a Hydro EV wet dispersion unit, a red He–Ne laser (λ = 633 nm), and a blue LED light source (λ = 470 nm). The stirring rate was set at 1500 rpm, and the volume-average particle diameter was calculated by averaging over five measurements.

### Optical and Fluorescence Microscopy

Representative images of the initial coarse phenanthrene crystals, the final phenanthrene microparticles, and the impact craters formed when firing phenanthrene microparticles at an aluminum foil target using a light gas gun were recorded using a Zeiss Axio Scope A1 microscope equipped with a Zeiss Axio ICm1 camera. Bright-field images of impact craters were recorded in reflectance mode using two LED light sources (N*ÄVLINGE* IKEA lamps) to illuminate the sample area. Fluorescence images were obtained using an LED radiation source combined with either filter set 02 (excitation λ = 365 nm, emission λ > 420 nm) or filter set 38 (excitation λ = 470 nm, emission λ = 525 nm).

### Scanning Electron Microscopy

Representative SEM images of the phenanthrene microparticles were recorded using an FEI Inspect-F instrument operating at an accelerating voltage of 10 kV and a beam current of 200 nA. Each sample was dried onto a glass slide before being sputter-coated by a thin overlayer of gold to prevent sample charging.

After each light gas gun experiment, the aluminum foil target was analyzed to identify the impact craters produced by the impinging phenanthrene microparticles. In this case, a Hitachi S3400N SEM instrument was used at an accelerating voltage of 10 kV and a beam current of 83 μA. An Oxford Instruments X-Max 80 mm^2^ energy-dispersive X-ray spectroscopy (EDX) detector was employed to map elemental compositions across individual impact craters. Carbon map images were recorded at four different angles, normalized with respect to brightness, and then merged to form a composite image. It is emphasized that the aluminum foil target was not gold-coated for these impact crater studies.

### Light Gas Gun Experiments

A two-stage light gas gun^14^ was employed for a “buckshot” experiment in which spherical phenanthrene microparticles of 25 μm diameter (prepared according to Scheme 1) were fired at an aluminum token target (40 mm × 40 mm × 1.48 mm; see Figure S2) covered by aluminum foil (grade Al1100) with a mean thickness of 100 μm. This aluminum foil target was positioned in the blast tank, and the gun was evacuated to a pressure of 0.44 mbar prior to firing. The impact velocity was determined to be 1.87 km s^–1^ (±0.5%), which is the same speed as that used in our prior study of non-spherical anthracene microparticles.^11^ A control experiment was also performed in the absence of any phenanthrene microparticles to enable assessment of the extent of target contamination (e.g., by gunpowder or gas residues, sabot fragments, etc.) during a shot. In this case, the light gas gun was fired at 1.74 km s^–1^ (±0.5%). In a second experiment, polypyrrole-coated 25 μm phenanthrene microparticles (mean polypyrrole overlayer thickness = 30 nm) were fired into a silica aerogel block (density = 32 ± 3 kg m^–3^) at 2.07 km s^–1^. These aerogel blocks are similar to those reported by Jones et al.^26^ In principle, such ultralow density targets enable capture of minimally processed microparticles even for impacts occurring within the hypervelocity regime.^27^

### FT-IR Spectroscopy Studies

FT-IR spectra were recorded for coarse phenanthrene crystals, polypyrrole bulk powder, polypyrrole-coated phenanthrene microparticles, and the insoluble black residue remaining after acetone extraction of polypyrrole-coated phenanthrene microparticles using a Perkin-Elmer Spectrum Two FT-IR spectrometer equipped with a UATR sampling accessory. Each spectrum was averaged over 20 scans and the spectral resolution was 2 cm^–1^.

### X-ray Photoelectron Spectroscopy

Phenanthrene, 25 μm diameter phenanthrene microparticles, polypyrrole-coated phenanthrene microparticles, and polypyrrole bulk powder were analyzed using a Kratos Axis Supra X-ray photoelectron spectrometer. In each case, powders were placed on indium foil, and spectra were recorded for two separate areas. Step sizes of 0.50 and 0.05 eV were used to record the survey spectra and high-resolution spectra, respectively. Casa XPS software (UK) was used to analyze the X-ray photoelectron spectroscopy (XPS) data.

## Results and Discussion

Recently, we reported that wet ball milling enables the convenient preparation of a concentrated aqueous suspension comprising relatively fine anthracene microparticles with a volume-average diameter of around 4 μm.^11^ However, this processing route involves the repeated random fracturing of macroscopic anthracene crystals, which inevitably leads to an ill-defined non-spherical morphology (see Figure 1b). In contrast, the relatively low melting point exhibited by phenanthrene was expected to facilitate the preparation of spherical microparticles via the “molten oil” route shown in Scheme 1. The latter morphology is particularly suitable for hypervelocity impact experiments because it simplifies analysis of the resulting impact craters.^28^ Typical SEM images recorded for such microparticles are shown in Figure 2, along with two representative particle size distributions obtained by using laser diffraction. Clearly, the phenanthrene microparticles obtained via the “molten oil” route exhibit a polydisperse spherical morphology. Moreover, the mean diameter can be readily adjusted by varying the emulsification conditions. For example, employing a stirring rate of 9000 rpm produced relatively large phenanthrene microparticles (Figure 2b) with a volume-average diameter of around 118 ± 50 μm (see Figure 2c). However, using a faster stirring rate of 18,000 rpm produced a volume-average diameter of approximately 25 ± 21 μm. We focus on the latter microparticles in the current study.

**Figure 3:**
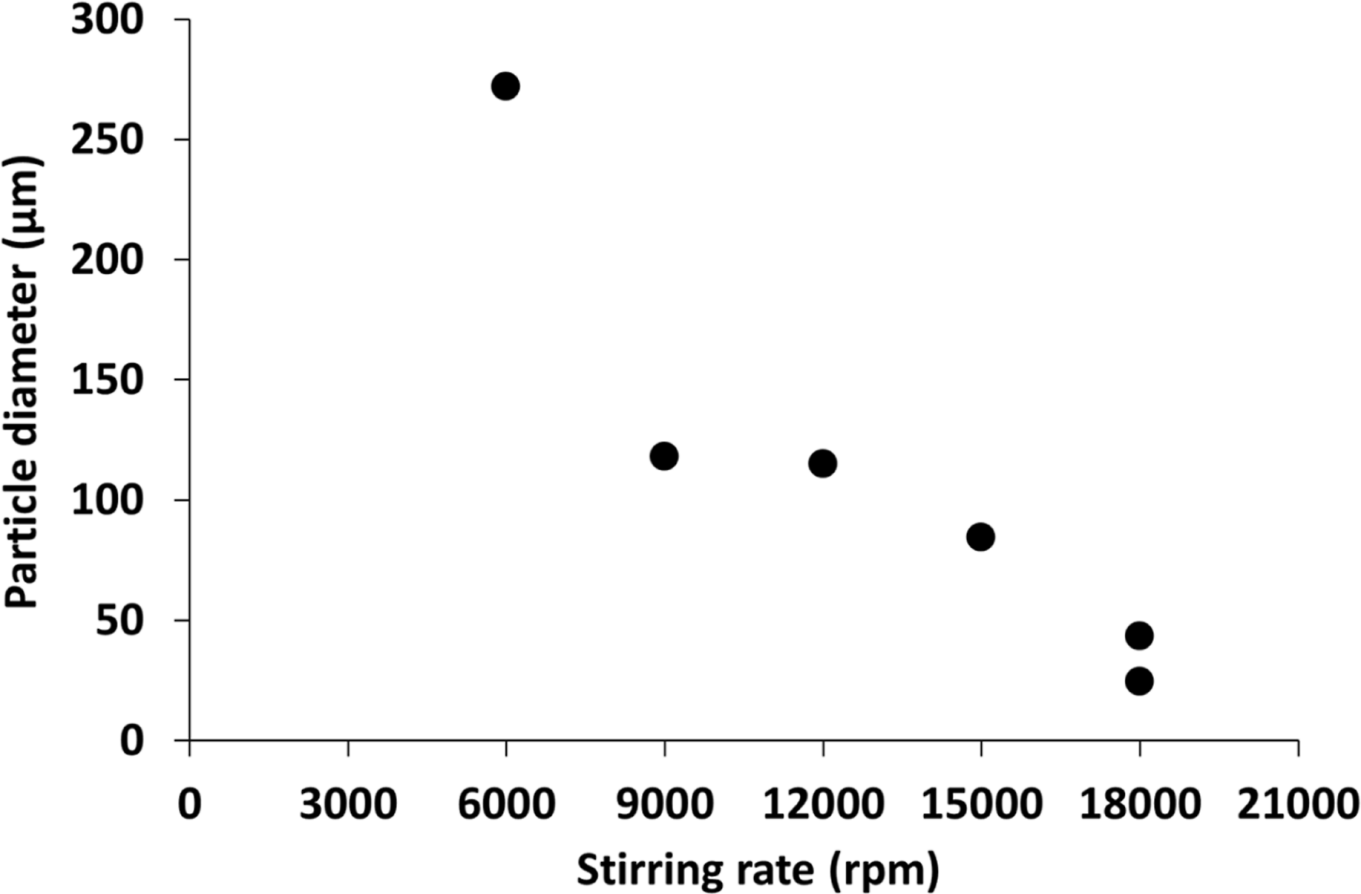
Effect of varying the stirring rate on the volume-average diameter of phenanthrene microparticles prepared via high-shear homogenization using the “hot emulsification” route outlined in Scheme 1.

The inverse relationship between the mean microparticle diameter and stirring rate is shown in Figure 3. Similar findings were reported by Bakhbakhi et al. when preparing phenanthrene microparticles using CO_2_ gas as an antisolvent.^29^

Additional SEM images recorded for microparticles prepared at a stirring rate of either 6000 or 12,000 rpm are shown in Figure S3, along with the corresponding particle size distributions obtained using laser diffraction. Representative optical microscopy images recorded for phenanthrene microparticles when the stirring rate is systematically varied are shown in Figure S4.

It is perhaps worth emphasizing that the hot emulsion or suspension should be filtered immediately to remove the continuous phase. Otherwise, a secondary population of non-spherical phenanthrene crystals is invariably formed in addition to the target spherical phenanthrene microparticles (see Figure S5). This problem is attributed to a reduction in the background solubility of phenanthrene within the continuous phase on cooling from 106 to 20 °C. Hot filtration removes this soluble fraction, which in turn minimizes secondary nucleation.

**Figure 4:**
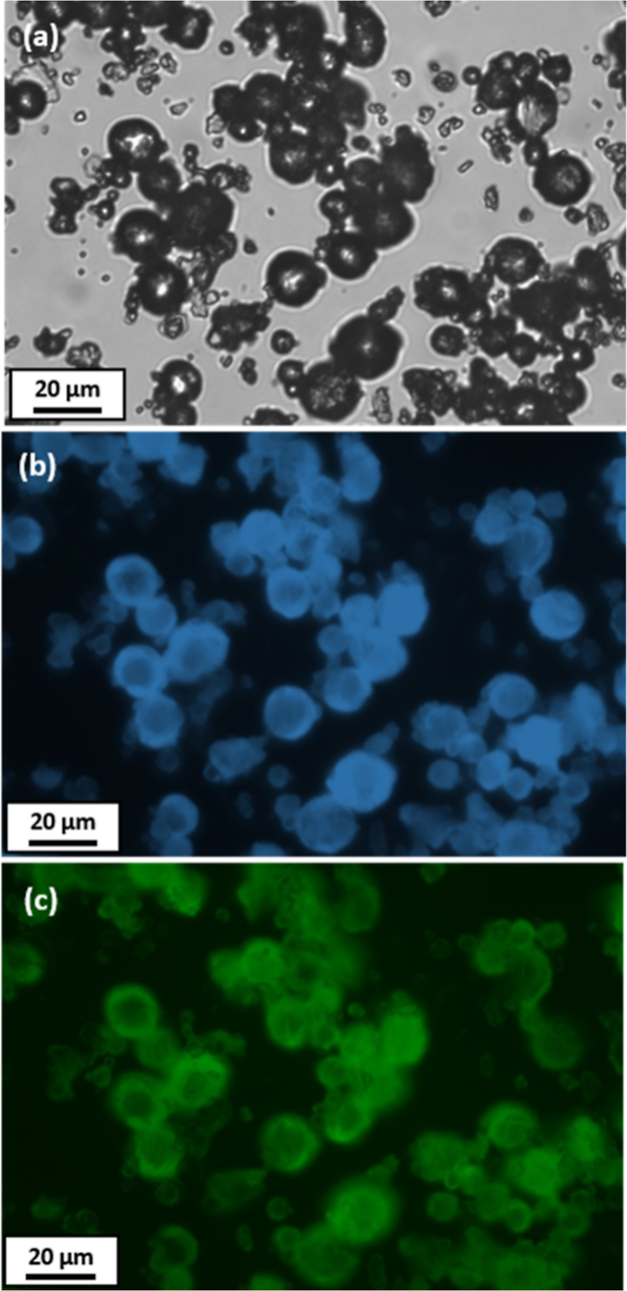
Representative images recorded for phenanthrene microparticles prepared via emulsification of molten phenanthrene in a 3:1 v/v water/ethylene glycol mixture at 106 °C: (a) optical microscopy, (b) fluorescence microscopy (filter set 02; excitation λ = 365 nm; emission λ > 420 nm), and (c) fluorescence microscopy (filter set 38; excitation λ = 470 nm; emission λ = 525 nm).

One interesting intrinsic chemical property of phenanthrene is its autofluorescence.^30^ Indeed, this well-known phenomenon has been used to detect a series of PAHs, which are widely regarded as undesirable environmentally persistent pollutants on Earth.^30−32^ For example, autofluorescence has enabled the migration of phenanthrene, anthracene, or pyrene within the soil to be monitored via confocal microscopy.^32^Figure 4 shows a second optical microscopy image recorded for phenanthrene microparticles prepared via high-shear homogenization. In this case, the corresponding fluorescence microscopy images recorded using an LED source are also shown in Figure 4b,c. The former image was obtained using filter set 02 (excitation λ = 365 nm, emission λ > 420 nm) while the latter image was obtained using filter set 38 (excitation λ = 470 nm; emission λ = 525 nm). Clearly, these phenanthrene microparticles exhibit autofluorescence in both cases.

Herein we examine whether autofluorescence is useful for assessing the chemical composition of the impact craters that are formed when firing such phenanthrene microparticles at metal targets using a light gas gun.^14^ In a control experiment, it was confirmed that the various other components (e.g., gun powder, sabot or burst disk fragments, etc.) that may contaminate the target when firing the light gas gun exhibit minimal autofluorescence (see Figure S2). Moreover, according to Chacko and co-workers, the UV-induced chemical degradation of phenanthrene produces relatively small nonaromatic molecular/atomic fragments that are unlikely to exhibit any autofluorescence.^25^ Thus, if autofluorescence is observed in the vicinity of impact craters, this should be a useful signature for phenanthrene survival after impact. In principle, this is a significant improvement on the analytical X-ray mapping capability used in conjunction with SEM analysis reported in our prior study.^11^ This is because the latter approach can readily identify carbonaceous debris but cannot distinguish among its various possible physical forms. For example, a carbon signal could simply indicate the presence of carbon char rather than intact phenanthrene.

**Figure 5:**
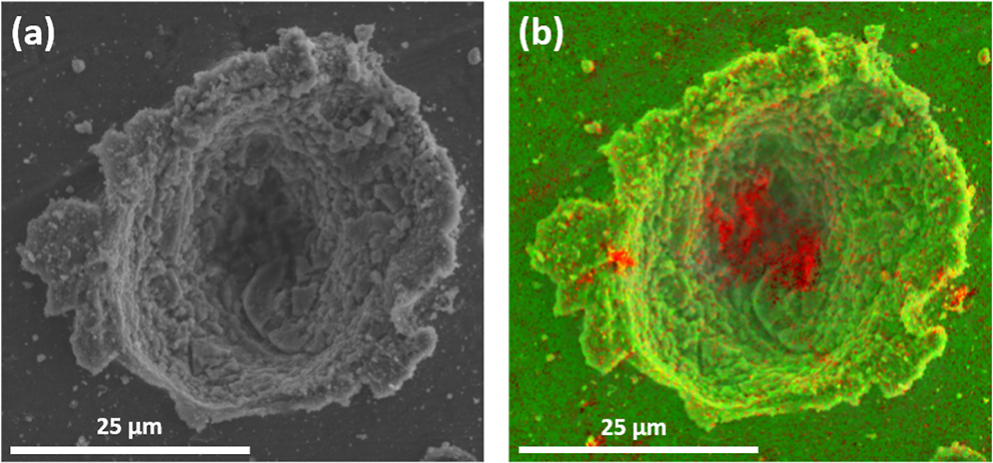
(a) Representative SEM image recorded for impact craters formed within an aluminum foil target after firing phenanthrene microparticles at 1.87 km s^–1^ using a two-stage light gas gun. (b) Representative elemental map of the SEM image shown in (a) recorded using EDX spectroscopy showing the presence of carbon debris (red pixels).

**Figure 6:**
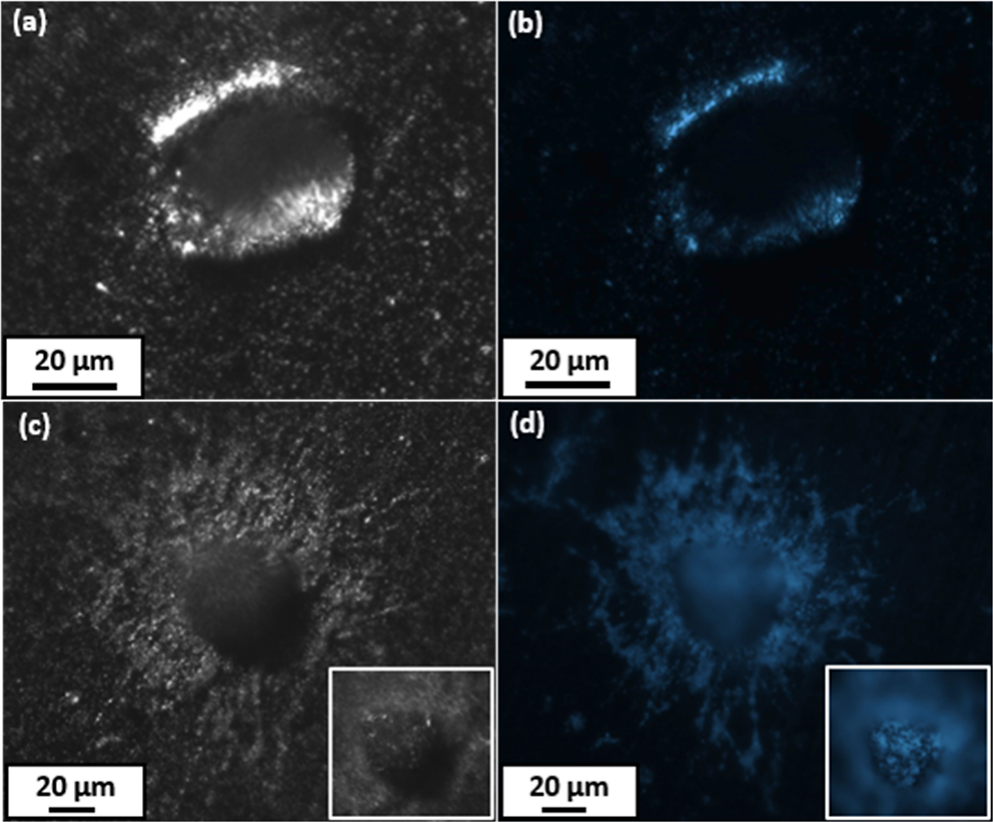
Optical microscopy images (a,c) and corresponding fluorescence microscopy images (b,d) recorded for two representative impact craters found in an aluminum foil target after firing phenanthrene microparticles at 1.87 km s^–1^ using a light gas gun. Insets shown in panels (c,d) were recorded using a different depth of focus to show the respective crater floors more clearly.

Figure 5a shows an SEM image recorded for a representative impact crater observed at the surface of an aluminum foil target after firing phenanthrene microparticles at 1.87 km s^–1^ using a two-stage light gas gun. The corresponding carbon elemental map for this impact crater obtained by using EDX spectroscopy is shown in Figure 5b. The latter image confirms the presence of carbonaceous debris (red pixels) associated with this crater, but its precise chemical form is not known. For comparison, pairs of optical microscopy and fluorescence microscopy images recorded for two further impact craters are shown in Figure 6. Inspecting images (a) and (b), autofluorescent debris corresponding to phenanthrene is clearly visible around the crater rim. Hence there is no doubt that at least some of this relatively delicate low molecular weight crystalline compound can survive a hypervelocity impact when striking a metal target. Inspecting images (c) and (d), autofluorescent debris is visible both in the vicinity of the crater and within the crater itself (see insets). In this case, the depth of focus of our optical microscope is insufficient to provide well-resolved images for the top and bottom of the crater simultaneously. Thus, the main image is focused on the crater rim and surrounding surface, while the corresponding inset image is focused on the crater floor.

In principle, the peak shock pressure during an impact can be estimated using the planar impact approximation.^33^ This calculation requires the shock Hugoniot coefficients of the linear wave speed equation for both the target and the projectile plus the initial density (ρ) for the target and the projectile.

The linear wave speed relationship is given by eq 1.
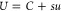
1where *U* is the shock speed, u is the particle speed, and *C* and *s* are the relevant shock Hugoniot coefficients (where *C* has units of m s^–1^ and *s* is a dimensionless quantity). Using literature data^34^ for the aluminum foil target (ρ = 2712 kg m^–3^) used in the present study gives *C* = 5376 ± 54 m s^–1^ and *s* = 1.339 ± 0.045. Fitting data reported by Marsh^34^ give *C* = 3139 ± 172 m s^–1^ and *s* = 0.309 ± 0.014 for phenanthrene (ρ = 1212 kg m^–3^). Assuming that the planar impact approximation is valid, we calculate a peak shock pressure of 6.4 GPa for the impact of phenanthrene on aluminum at an impact speed of 1.87 km s^–1^.

Assuming that no phase change occurs, Artemieva and Ivanov^35^ used shock Hugoniot coefficients to demonstrate that the peak post-shock elevated temperature (*T*_ps_) in the most heavily shocked part of the projectile can be estimated using eq 2.
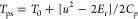
2where *C*_*p*_ is the specific heat capacity, *T*_0_ is the initial (room) temperature, and *E*_r_ is the energy lost from the projectile during release from its shocked state. *E*_r_ is given by eq 3.
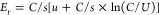
3

This approach has been used to analyze impacts on aluminum foil for various types of microparticles, including pyrrhotite,^36^ olivine and diopside,^37^ calcite,^38^ and salt.^39^

Assuming *C*_*p*_ = 220.6 J mol^–1^ K^–1^^40^ and taking ambient temperature to be 293 K, an impact speed of 1.87 km s^–1^ produces a *T*_ps_ value of 96 ± 2 °C, which is just below the melting point for phenanthrene (101 °C). This is consistent with the implicit assumption that the high-energy impact does not induce any phase change in the projectile material. A further assumption is that the energy density for such impacts is not dependent on the particle mass or size. In this context, it is known that strain rate strength effects become important for smaller particles (i.e., at the micron length scale or below) traveling at more than a few km s^–1^, which changes the shock Hugoniot coefficients and thus the peak pressures and temperatures.^41^

**Scheme 2:**
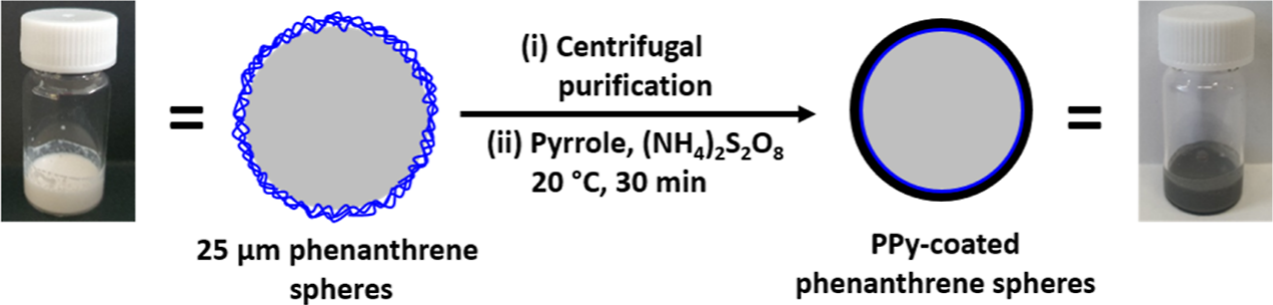
Schematic Cartoon for the Preparation of Polypyrrole-Coated Phenanthrene Microparticles Using a Well-Known Aqueous Deposition Protocol for the Polymerization of Pyrrole^43^

It is well known that polypyrrole can be readily deposited onto hydrophobic substrates from aqueous solution.^11,42−44^ For example, the polymerization of pyrrole in the presence of 1–2 μm diameter sterically-stabilized polystyrene latex particles using an FeCl_3_ oxidant leads to well-defined polypyrrole-coated polystyrene latexes.^43^ Indeed, such microparticles are excellent synthetic mimics for carbon-rich cosmic dust because they can be accelerated up to 10–20 km s^–1^ using a high-voltage van de Graaff accelerator.^14^ Similarly, polypyrrole-coated polystyrene latex particles of 20 μm diameter have been fired into aerogel targets at up to 6 km s^–1^ using a light gas gun. In this case, the ultrathin polypyrrole coating served as a sacrificial overlayer that enabled the extent of thermal ablation during aerogel capture to be assessed.^45^ Given their similar dimensions, we decided to coat the 25 μm diameter phenanthrene microparticles by targeting a mean polypyrrole overlayer thickness of approximately 30 nm by using the aqueous deposition protocol summarized in Scheme 2. The mean polypyrrole shell thickness, *x*, can be calculated using eq 4.
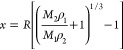
4where *R* is the mean radius of the phenanthrene microparticles, *M*_2_ and *M*_1_ are the mass fractions of the polypyrrole shell and phenanthrene core components, and ρ_2_ and ρ_1_ are the densities of polypyrrole and phenanthrene, respectively.

We chose to use (NH_4_)_2_S_2_O_8_ for the oxidative polymerization of pyrrole. This is because preliminary experiments conducted with FeCl_3_ unexpectedly indicated partial loss of the spherical morphology of the phenanthrene microparticles. It is known that PAHs such as naphthalene, anthracene, or pyrene undergo oxidative polymerization on heating in the presence of FeCl_3_.^46^ Presumably, phenanthrene undergoes a similar side reaction, which leads to a change in its morphology. Fortunately, no such problems were encountered when using (NH_4_)_2_S_2_O_8_, which has been previously used to prepare polypyrrole-coated pyrrhotite and polypyrrole-coated olivine microparticles.^47,48^ This oxidant leads to a much faster rate of pyrrole polymerization than FeCl_3_ but a slightly lower conductivity.^49,50^ Thus, it can be assumed that (i) all of the pyrrole monomer is converted into polypyrrole and (ii) all of the polypyrrole is deposited onto the phenanthrene microparticles (i.e., there is no secondary nucleation).^43^ These assumptions have been validated when coating 20 μm diameter polystyrene latex particles with a polypyrrole coating of comparable mean thickness (20 nm).^45^

**Figure 7:**
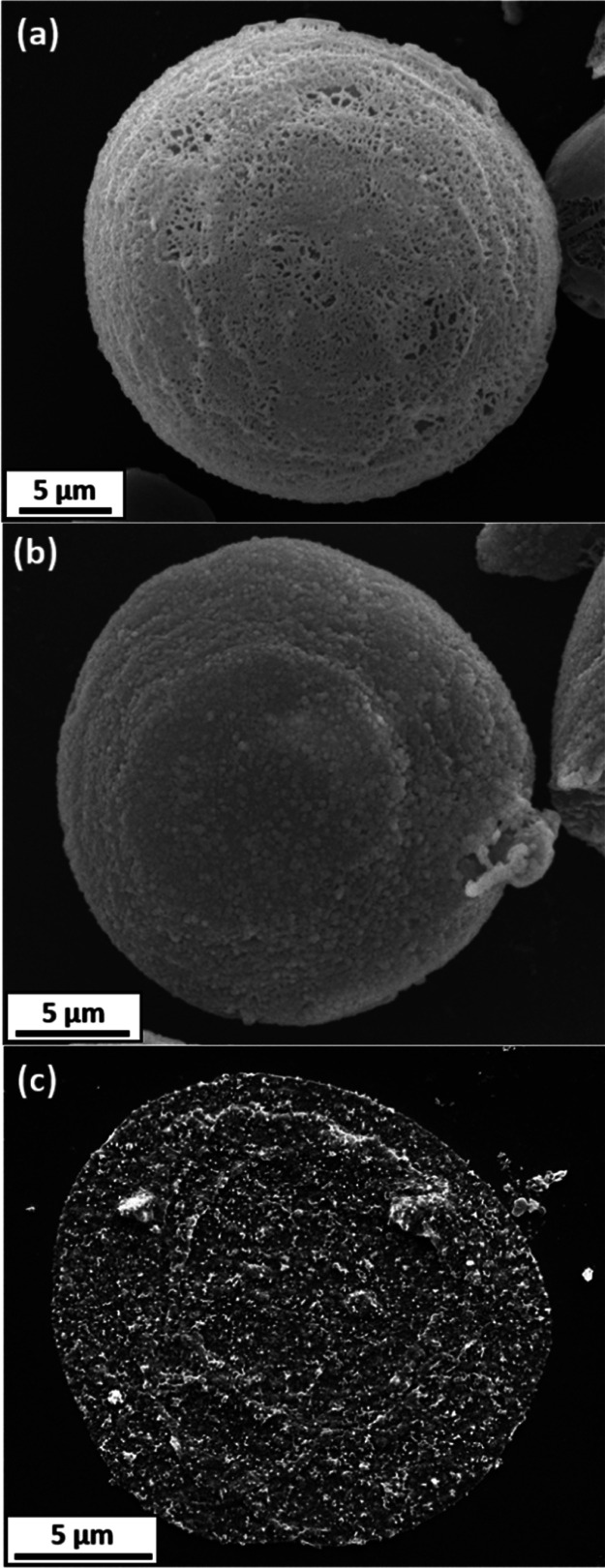
High magnification SEM images recorded for: (a) an individual phenanthrene microparticle; (b) a polypyrrole-coated phenanthrene microparticle; (c) the black insoluble residue obtained after solvent extraction of the underlying phenanthrene core using acetone.

High-magnification SEM images were recorded for an individual phenanthrene microparticle and a polypyrrole-coated phenanthrene microparticle (Figure 7a,b). A comparison of these two images confirms a subtle change in the surface morphology: deposition of the polypyrrole overlayer leads to a distinctive globular morphology that is characteristic of this conducting polymer.^41^Figure 7c shows an SEM image recorded for the black insoluble residue that remains after solvent extraction using acetone, which is a good solvent for the underlying phenanthrene core.

Noting the strikingly similar dimensions (see scale bars), this image clearly depicts a collapsed (rather than a free-standing) polypyrrole shell. However, spectroscopic evidence that such residues indeed correspond to polypyrrole is still required.

**Figure 8:**
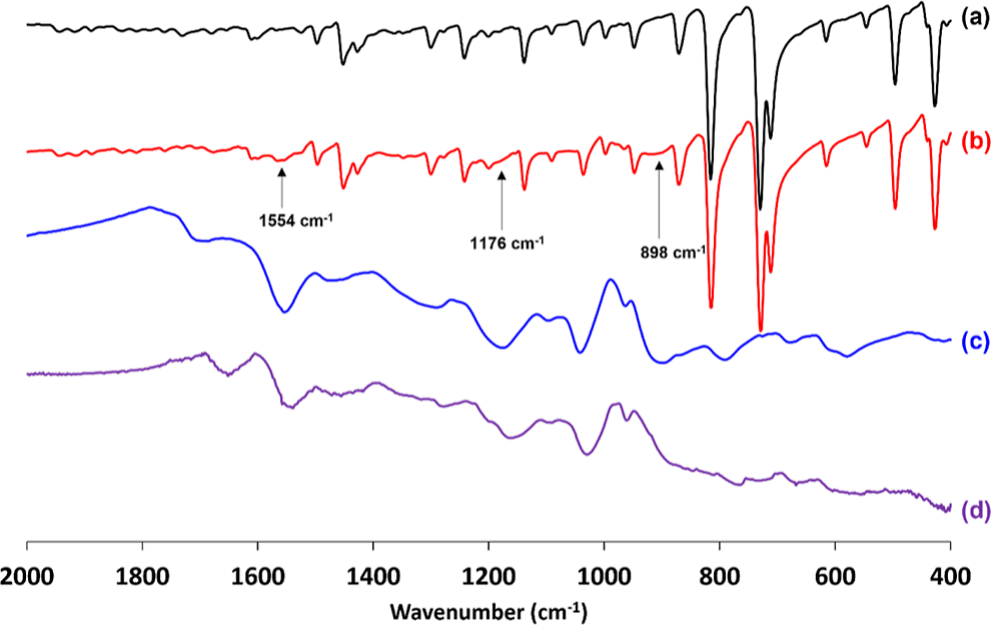
FT-IR spectra recorded for (a) 25 μm diameter phenanthrene microparticles, (b) the corresponding polypyrrole-coated phenanthrene microparticles (target polypyrrole overlayer thickness = 30 nm), (c) polypyrrole bulk powder, and (d) the black insoluble residues that remains after solvent extraction of phenanthrene from the polypyrrole-coated phenanthrene microparticles using acetone.

Accordingly, Figure 8 shows FT-IR spectra recorded for 25 μm diameter phenanthrene microparticles, polypyrrole-coated phenanthrene microparticles, and polypyrrole bulk powder. In addition, a spectrum was recorded for the insoluble residues that remained after solvent extraction of the underlying phenanthrene from the polypyrrole-coated phenanthrene microparticles using acetone. The FT-IR spectrum obtained for the 25 μm diameter phenanthrene microparticles compares well with that reported for phenanthrene in the literature.^51^ For example, prominent sharp bands at 731 and 814 cm^–1^ correspond to out-of-plane aromatic C–H bending modes, whereas the less intense bands at 1240 and 1450 cm^–1^ correspond to C–H in-plane bending and C–C stretch, respectively. Similarly, the spectrum recorded for polypyrrole bulk powder is representative, with strong broad bands observed at 1554, 1458, 1176, 1042, and 898 cm^–1^ being characteristic of the electrically conductive form of this material.^50^ The FT-IR spectrum obtained for the polypyrrole-coated phenanthrene microparticles resembles that of pure phenanthrene, with minimal evidence for the polypyrrole overlayer. This is perhaps not surprising given that the polypyrrole content of such microparticles is only approximately 0.9% by mass. The black insoluble material remaining after acetone extraction has an FT-IR spectrum that is almost identical to that of polypyrrole bulk powder. Combined with the SEM image shown in Figure 7c, this provides strong evidence for the contiguous nature of the original polypyrrole overlayer.

**Figure 9:**
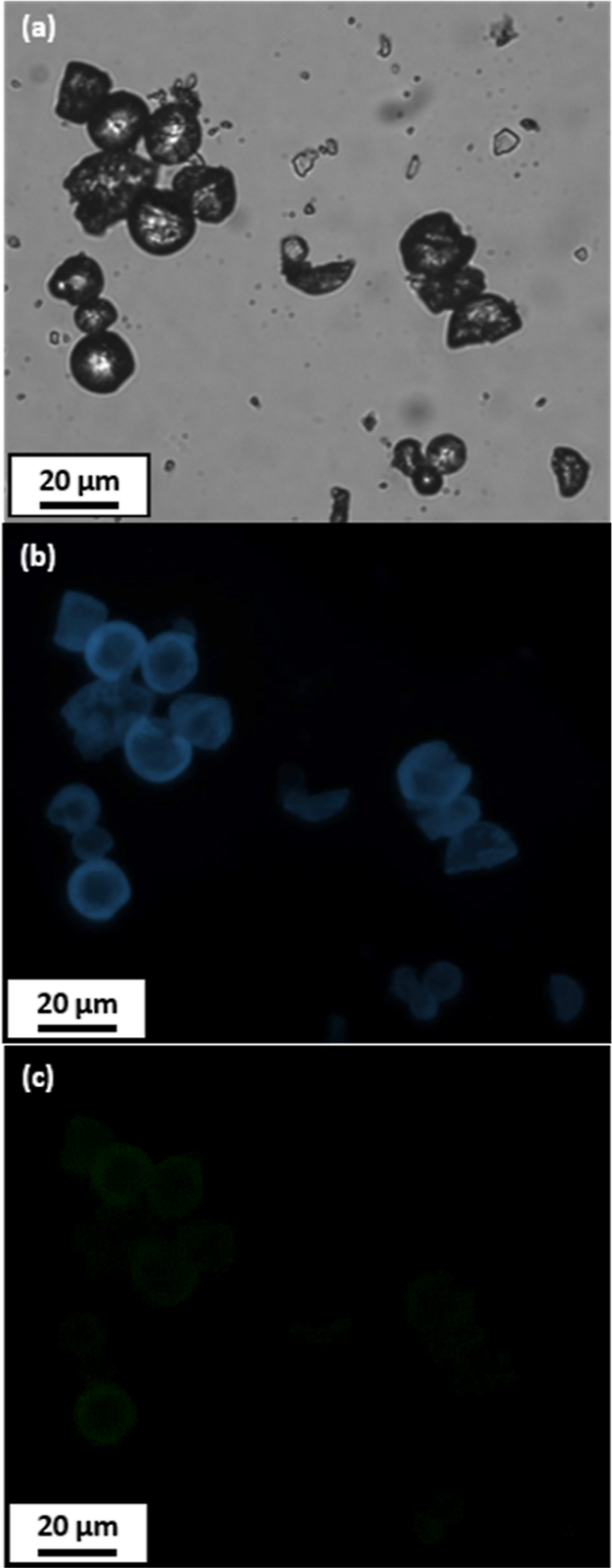
(a) Optical microscopy image recorded for polypyrrole-coated phenanthrene microparticles. (b) Corresponding fluorescence microscopy image recorded for polypyrrole-coated phenanthrene microparticles (excitation wavelength = 365 nm, emission wavelength > 420 nm). (c) Corresponding fluorescence microscopy image recorded for polypyrrole-coated phenanthrene microparticles (excitation wavelength = 470 nm, emission wavelength > 525 nm). Comparing the latter two images, the fluorescence arising from the underlying phenanthrene cores can be either readily visualized (shorter excitation wavelength) or partially obscured by the strongly absorbing polypyrrole overlayer (longer excitation wavelength).

An optical microscopy image recorded for the polypyrrole-coated phenanthrene microparticle is shown in Figure 9a. The corresponding image recorded for the same microparticles after switching to the fluorescence microscopy mode is shown in Figure 9b. The intrinsic autofluorescence associated with the underlying phenanthrene cores is clearly revealed under the chosen experimental conditions (e.g., filter set 02; excitation λ = 365 nm, emission λ > 420 nm). Presumably, this relatively short excitation wavelength is sufficient to penetrate the ultrathin overlayer of strongly absorbing polypyrrole. The corresponding image recorded for the same microparticles when using an alternative set-up (e.g., filter set 38; excitation λ = 470 nm, emission λ = 525 nm) is shown in Figure 9c. Comparing this image with that shown in Figure 9b, it is evident that the strongly absorbing polypyrrole shells efficiently obscure the fluorescence associated with the underlying phenanthrene cores when employing a longer excitation wavelength. This is interesting, not least because it provides useful additional supporting evidence for the polypyrrole overlayer.

**Figure 10:**
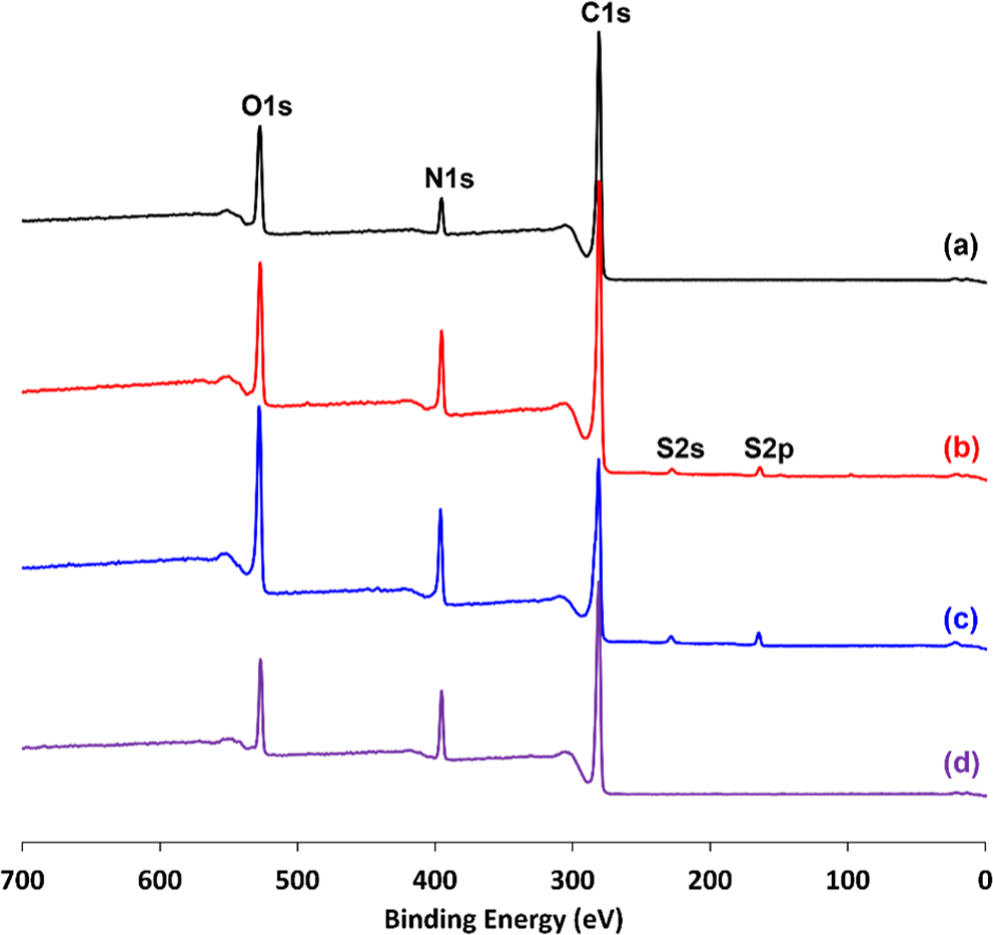
X-ray photoelectron survey spectra recorded for (a) 25 μm phenanthrene microparticles, (b) polypyrrole-coated 25 μm phenanthrene microparticles (target polypyrrole overlayer thickness = 30 nm), (c) polypyrrole bulk powder, and (d) poly(*N*-vinylpyrrolidone).

X-ray photoelectron survey spectra recorded for 25 μm phenanthrene microparticles and polypyrrole-coated 25 μm phenanthrene microparticles (target polypyrrole overlayer thickness = 30 nm), polypyrrole bulk powder, and poly(*N*-vinylpyrrolidone) are shown in Figure 10.

Pure phenanthrene (C_14_H_10_) contains no nitrogen atoms and the XPS sampling depth is typically 2–10 nm.^52^ Thus, the observation of a weak N1s signal for the phenanthrene microparticles is attributed to the surface adsorption of the poly(*N*-vinylpyrrolidone) emulsifier. Comparing the intensity of this N1s signal to that observed for poly(*N*-vinylpyrrolidone) suggests a surface coverage of around 47%. The much stronger N1s signal observed for the polypyrrole-coated phenanthrene microparticles is (mainly) attributed to the polypyrrole overlayer. This interpretation is consistent with the target polypyrrole overlayer of 30 nm, which exceeds the XPS sampling depth. Moreover, the observation of additional S2s and S2p signals enables calculation of an S/N atomic ratio of approximately 0.14. Essentially the same S/N atomic ratio was determined for the polypyrrole bulk powder control. This is consistent with the electrically conductive form of polypyrrole and suggests that the intercalated counterions are likely to be SO_4_^2–^.^53^

**Figure 11:**
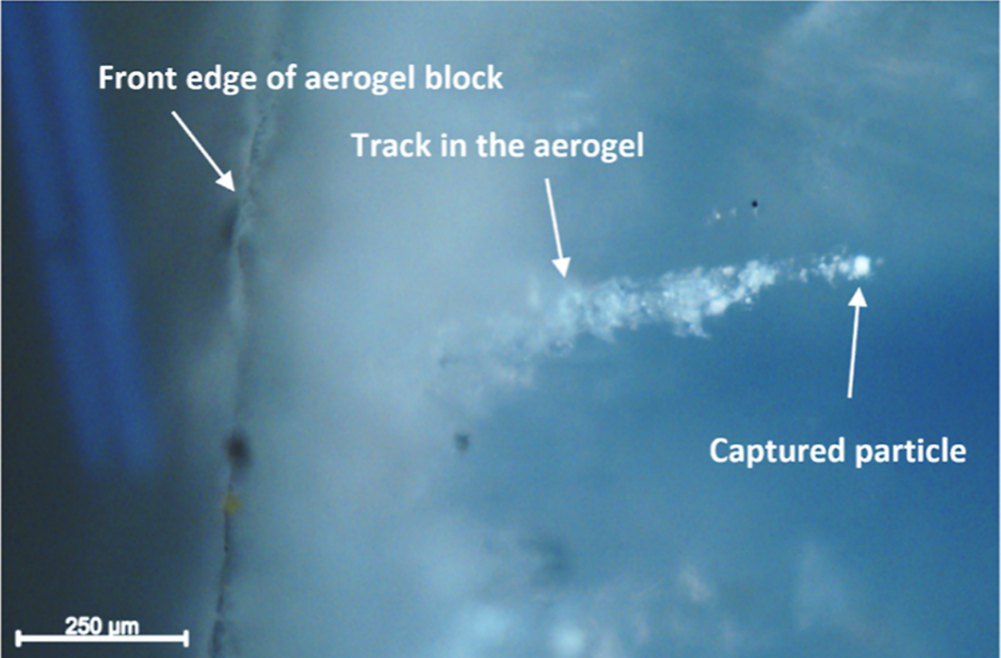
Optical microscopy image (side view) recorded after firing 25 μm diameter polypyrrole-coated phenanthrene microparticles into an aerogel target at 2.07 km s^–1^. An individual microparticle has entered the target from the left and formed a distinctive “carrot track” prior to its capture.

Based on our prior study,^45^ we envisaged that these new polypyrrole-coated phenanthrene microparticles should be useful for assessing the extent of thermal ablation suffered by PAH-rich cosmic dust during their capture within an ultralow density aerogel target. Accordingly, 25 μm diameter polypyrrole-coated phenanthrene microparticles were fired into an aerogel target at 2.07 km s^–1^ using the light gas gun. Optical microscopy studies of the aerogel target revealed the presence of multiple characteristic “carrot tracks” within the aerogel, with individual microparticles located at the end of each track (see Figure 11).

The peak shock pressure generated during such an aerogel impact can be calculated using the planar impact approximation. The C and S values for aerogel were taken from Anderson,^54^ who suggests that *C*/km s^–1^ = 0.436 – 2.024·ρ + 4.18·ρ^2^ and *S* = 0.700 + 24.4·ρ – 36.3·ρ^2^, where ρ is the aerogel density in mg m^–3^. These two equations yield *C* = 0.375 km s^–1^ and *S* = 1.443 for the aerogel target studied herein, which indicates that the peak shock pressure on impact is approximately 0.212 GPa. As expected, this is much lower than the impact on the aluminum foil target at a comparable hypervelocity, which in turn leads to a minimal increase in local temperature for the rapidly decelerating microparticles during their capture. Nevertheless, thermal ablation may still result in some degree of surface processing of the microparticles during their passage through the aerogel.

**Figure 12:**
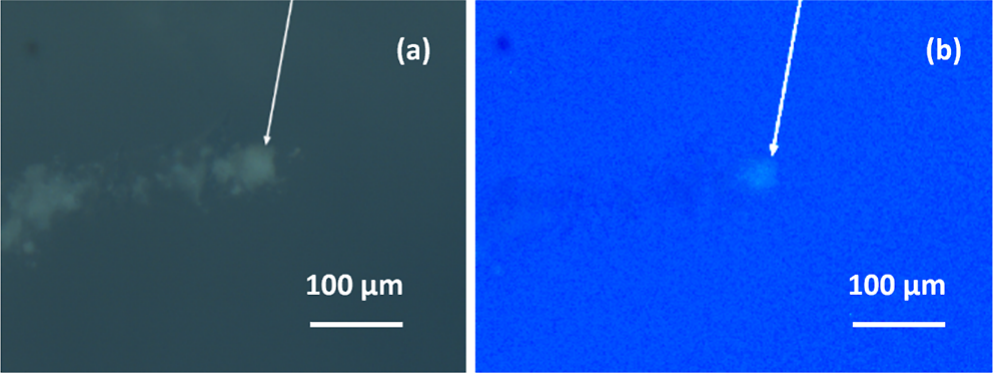
An individual microparticle (see white arrow) captured intact within an aerogel target illuminated using (a) visible light and (b) a UV lamp. This microparticle entered from the left-hand side and its characteristic carrot track is discernible when viewed using visible light but not when using UV light. In contrast, the captured microparticle is visible when using either type of illuminating radiation.

Importantly, illumination of the aerogel target using a UV lamp (λ_max_ = 365 nm) enabled intrinsic autofluorescence for such captured microparticles to be observed (see Figure 12).

This suggests that the ultrathin polypyrrole overlayers experience at least some degree of thermal ablation to expose the underlying phenanthrene cores. In the case of the Stardust mission,^55^ this spacecraft’s encounter velocity of 6.1 km s^–1^ with comet 81P/Wild was most likely too high to prevent some degree of thermal processing/degradation of organic cometary dust grains captured within its aerogel targets. Nevertheless, IR spectroscopy studies confirmed characteristic aliphatic and aromatic bands as well as organic carbon.^2,56^ Moreover, several PAH species (e.g., naphthalene, phenanthrene, and pyrene) were detected along the inner walls of the carrot tracks.^2^ In principle, the new model core–shell microparticles reported herein should provide an opportunity to assess the extent of thermal processing that can occur as a function of impact speed when capturing PAH-based dust grains within aerogels. Such physical insights are expected to be invaluable for the appropriate design of future space missions, whose goal is to collect intact organic cometary dust grains for subsequent analysis (either in situ or on Earth). In principle, the same approach could be used to sample the icy plumes emanating from the Saturnian satellite, Enceladus.^22^

## Conclusions

We have exploited the low melting point of phenanthrene to prepare spherical microparticles via high-shear homogenization of the corresponding molten oil in a 3:1 water/ethylene glycol mixture using a commercial non-ionic polymeric emulsifier at 106 °C. Such microparticles are the first example of a synthetic mimic for PAH-based cosmic dust that exhibits a well-defined spherical morphology. A two-stage light gas gun is used to fire these model projectiles into an aluminum foil target at 1.87 km s^–1^ to produce impact craters. The autofluorescence exhibited by phenanthrene aids the analysis of such craters by fluorescence microscopy because it provides direct visual evidence for the survival of phenanthrene and indicates its spatial distribution both inside and outside each crater. Moreover, such phenanthrene microparticles can be coated with an ultrathin overlayer of polypyrrole, which significantly reduces their autofluorescence. In principle, such core–shell microparticles should be useful for assessing the extent of thermal ablation that is likely to occur when they are fired into aerogel targets. To examine this hypothesis, these microparticles were fired into an aerogel target at 2.07 km s^–1^. Weakly autofluorescent microparticles were observed at the ends of carrot tracks, which suggests that their phenanthrene cores survived this hypervelocity impact. Thus these new core–shell microparticles should be useful model projectiles for assessing the extent of thermal processing that may occur during aerogel capture. Such experiments would have important implications for future space missions that aim to capture PAH-based dust grains originating from cometary tails or to sample organic dust grains within plumes emanating from icy satellites such as Enceladus.

## Supporting Information Available

Schematic experimental set-up used for the preparation of phenanthrene microparticles; digital photograph, bright-field optical and fluorescence microscopy images recorded for an aluminum foil target after firing a blank shot (no projectile); additional SEM images and laser diffraction particle size distribution curves for phenanthrene microparticles prepared at 6000 rpm and 12,000 rpm; optical microscopy images recorded for phenanthrene microparticles formed when varying the stirring rate from 6000 to 18,000 rpm; and optical microscopy images of non-spherical phenanthrene crystals formed during aging of a suspension of spherical phenanthrene microparticles when cooling from 106 to 20 °C (PDF)
