# Supplementary material for: Synthesis of Autofluorescent Phenanthrene Microparticles via Emulsification: A Useful Synthetic Mimic for Polycyclic Aromatic Hydrocarbon-Based Cosmic Dust
Source: ACS Appl Mater Interfaces. 2023 Nov 9;15(46):54039–49. doi: 10.1021/acsami.3c08585 (PMC10685351; doi:10.1021/acsami.3c08585)
Supplement: Supplementary file 1 — am3c08585_si_001.pdf [file am3c08585_si_001.pdf]

Supporting Information

Synthesis of autofluorescent phenanthrene microparticles via emulsification: a useful synthetic mimic for polycyclic aromatic hydrocarbon-based cosmic dust

Derek H. H. Chan [a], Jessica L. Wills [c], Jon D. Tandy [b], Mark J. Burchell [c],

Penelope J. Wozniakiewicz [c], Luke S. Alesbrook[c] and Steven P. Armes [a,*]

a. Dainton Building, Department of Chemistry, University of Sheffield, Brook Hill, Sheffield, South Yorkshire, S3 7HF, UK.

b. School of Chemistry and Forensic Science, Centre for Astrophysics and Planetary Science, University of Kent, Canterbury, Kent, CT2 7NZ, UK.

c. School of Physics and Astronomy, Centre for Astrophysics and Planetary Science, University of Kent, Canterbury, Kent, CT2 7NH, UK.

Summary of Contents

Figure S1. Experimental set-up for the preparation of phenanthrene microparticles used in this study.

Figure S2. Digital photograph, brightfield optical and fluorescence microscopy images recorded for an aluminum foil target after firing a blank shot (no projectile).

Figure S3. SEM images and laser diffraction particle size distribution curves for phenanthrene microparticles prepared at 6,000 rpm and 12,000 rpm.

Figure S4. Optical microscopy images recorded for phenanthrene microparticles prepared by varying the stirring rate from 6,000 to 18,000 rpm.

Figure S5. Optical microscopy images of non-spherical phenanthrene crystals during ageing of a suspension of spherical phenanthrene on cooling from 106 °C to 20 °C.

* Author to whom correspondence should be addressed (s.p.armes@shef.ac.uk).

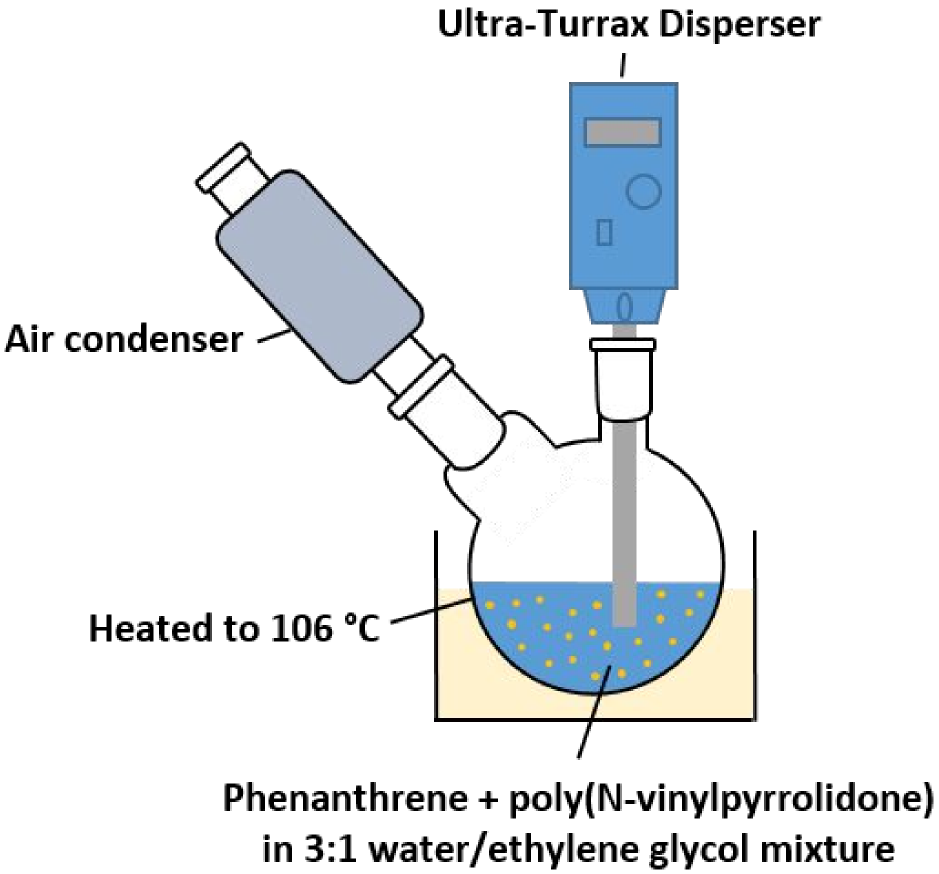

Figure S1. Experimental set-up for the preparation of phenanthrene microparticles via high-shear emulsification of molten phenanthrene in a 3:1 v/v water/ethylene glycol mixture at 106 °C.

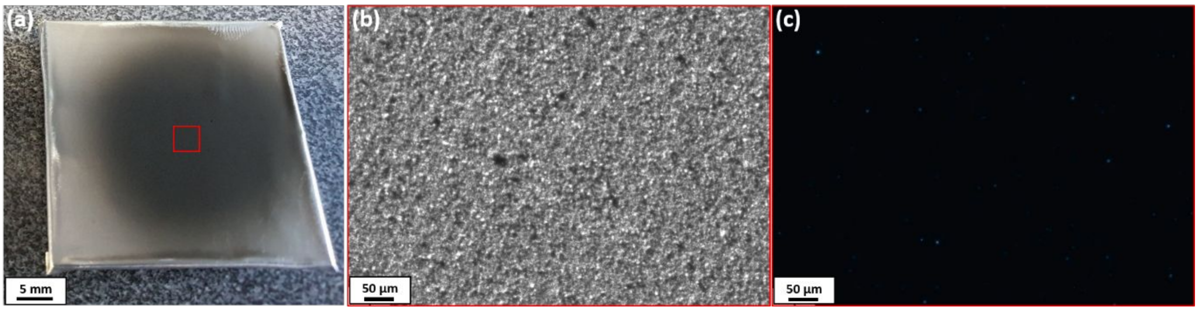

Figure S2. (a) Digital photograph recorded for an aluminum foil target showing an extensive dark grey area assigned to carbon residues after firing a blank shot (no projectile) at 1.74 km s^{-1} using a two-stage light gas gun. (b) Brightfield optical microscopy image and (c) corresponding fluorescence microscopy image recorded at the centre of the dark grey area (see red box) shown in image (a). This control experiment confirms that such carbon residues exhibit minimal autofluorescence. Thus any autofluorescence observed when firing the phenanthrene microparticles can be attributed to their survival after the hypervelocity impact.

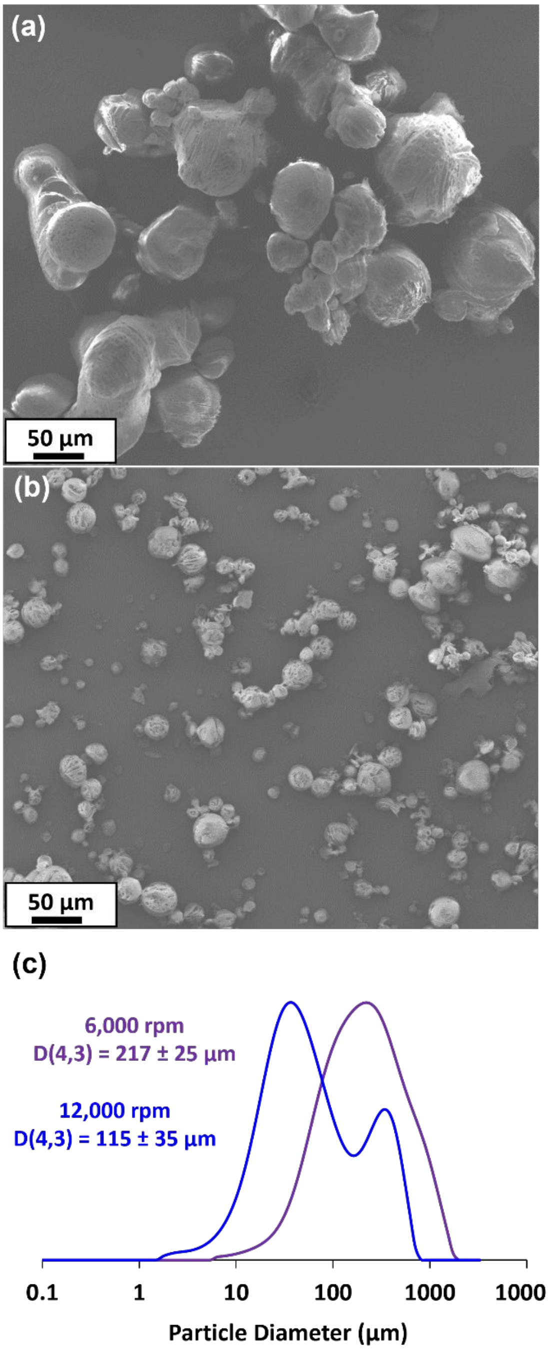

Figure S3. Representative scanning electron microscopy images obtained for phenanthrene microparticles prepared at (a) 6,000 rpm and (b) 12,000 rpm via high shear emulsification of molten phenanthrene. (c) Typical particle size distributions obtained for the same phenanthrene microparticles as determined by laser diffraction.

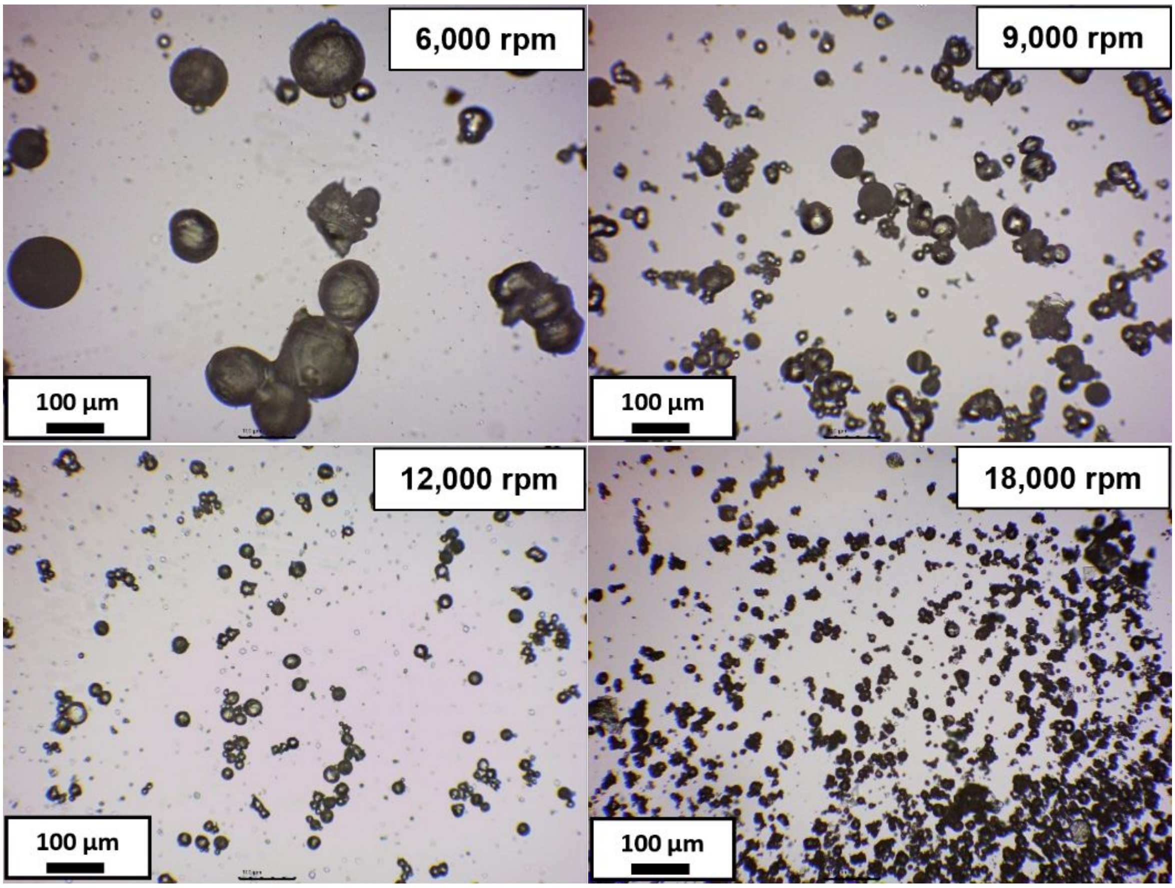

Figure S4. Representative optical microscopy images recorded for phenanthrene microparticles prepared by systematically varying the stirring rate from 6,000 to 18,000 rpm via high-shear emulsification of molten phenanthrene in a 3:1 v/v water/ethylene glycol mixture at 106 °C.

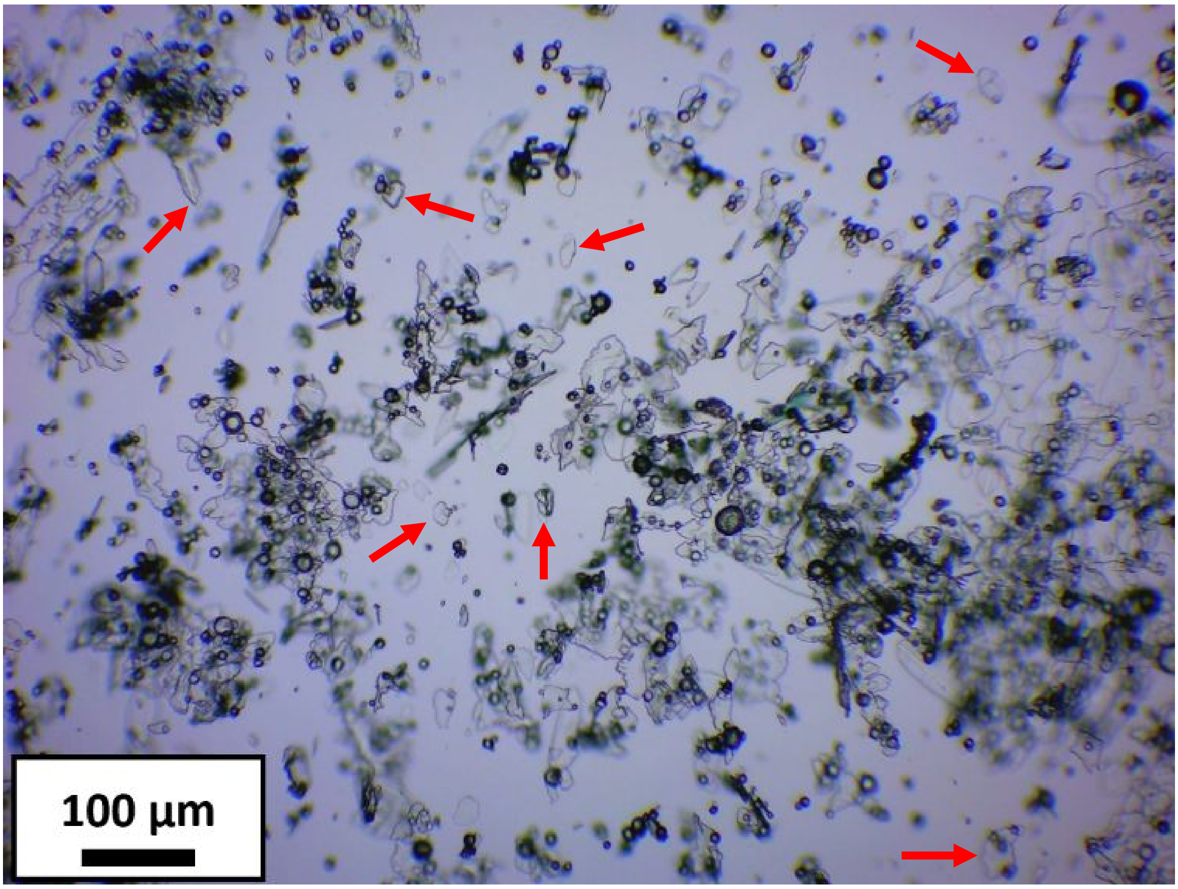

Figure S5. Representative low magnification optical microscopy image illustrating the formation of non-spherical phenanthrene crystals during ageing of a suspension of spherical phenanthrene microparticles in a 3:1 water/ethylene glycol mixture. This secondary phase (see red arrows) is attributed to the precipitation of a small amount of dissolved phenanthrene on cooling from 106 °C to 20 °C.
